# For Augustinian Archival Openness and Laggardly Sharing: Trustworthy Archiving and Sharing of Social Science Data From Identifiable Human Subjects

**DOI:** 10.3389/frma.2021.736568

**Published:** 2021-10-14

**Authors:** David Zeitlyn

**Affiliations:** Institute of Social and Cultural Anthropology, School of Anthropology and Museum Ethnography University of Oxford, Oxford, United Kingdom

**Keywords:** human subjects, research ethics, embargo, GDPR, archives/ archiving, pseudonymisation, conflicting responsibilities

## Introduction

Saint Augustine used to pray “Lord, make me chaste—but not yet.” This finds a parallel in qualitative social science where researchers may endorse data sharing and the calls for open science but with a hesitation that results from conflicting desires and obligations. How can one be open and share research material while respecting moral, ethical and legal requirements not to share? Many of the conflicts can be resolved by adding delay into the process: summarized in an Augustinian inspired archival prayer “to make me open—but not yet.” There is warrant for this but existing infrastructures may not easily accommodate it not so much because of technical problems but more because they are not resourced to plan over a long enough timescale.

Social science data involves other sorts of trust than those implicit in the call for contributions to the Frontiers Topic “Trust and Infrastructure in Scholarly Communications” (https://www.frontiersin.org/research-topics/18191/trust-and-infrastructure-in-scholarly-communications?utm_source=F-RTM&utm_medium=CFP_E2&utm_campaign=PRD_CFP_T1_RT-TITLE#overview and in statements promoting the idea of open data, for example, by the Open Data Institute RRID:SCR_021681 https://theodi.org/about-the-odi/). These are relevant to the design, implementation and funding of infrastructures supporting research beyond medical, chemical and physical disciplines. My areas of concern are qualitative social sciences as well as many humanities disciplines including oral history and life history research where, as a referee points out, “the tellers want their stories told” or the participants contribute because they want to contribute to the general development of science (Kuula, 2011). What is distinctive about these disciplines is that as an essential part of the research process, the researchers have human, interpersonal relationships with their interlocutors (which makes terms like informants, interviewees, research subjects or collaborators misleading if not inappropriate).^1^ Also much of the importance of the material gathered lies precisely in the aspects that are destroyed by anonymization. Writing as an anthropologist I talk about anthropology but the discussion is relevant to all those undertaking qualitative research with living humans, especially those where more or less structured interviews are not the main form of data collection.^2^

I think it helps to distinguish different types of trust since they need different sorts of archiving infrastructure. Discussions of data sharing address mainly the third of these.

**Table:** 

Trust type	Gloss	Infrastructural implications
Trust_1_ (T_1_)	Participants trust researcher	Long Term Embargos (census style)
Trust_2_ (T_2_)	Researchers trust participants	Readers can access data (data sharing)
Trust_3_ (T_3_)	Readers trust researcher	Readers can access data (data sharing)

As institutionalized by research ethics boards when a researcher starts talking to an “informant” a first step is to work through a consent process. As has been long pointed out (Neale and Bishop, 2012; Bell, 2014), for qualitative research subjects such as anthropology this is often inadequate and misleading but it continues, driven by forms of institutional inertia and what Wynn and lsrael (2018) describe as a *fetishization* of signed consent forms. Commonly, researchers promise to respect the confidentiality of what they have been told by anonymizing the individuals concerned. Until relatively recently this was taken (usually tacitly) to mean anonymizing *in publications* (strictly this is pseudonymisation since versions with identifiers were retained). The GDPR in the EU (and in UK) and its implementation by administrators in research ethics boards has raised the possibility of its application to the actual data collected on which publications are based (see Yuill, 2018) despite its explicit provisions for data archiving. For many researchers the “research material” (a more generous term than data) may be scrappy, poorly structured and may seem inadequate to others without the advantage of having taken part in the research process. For the original researcher they may be more *aide memoire* than hard data but not necessarily the worse for this. Sharing such material may not help assessment of publications based on it. With apologies for a double negative, this is *not* to say they are untrustworthy. Between researcher and reader there is an “ethnographic pact” (paralleling Philippe Lejeune’s “autobiographical pact” Lejeune, 1989).^3^ For all that sharing such material may have other benefits. It makes the material available for use by other researchers from other disciplines, and to those from the places where it may have originated. Usefulness/comprehensibility is not all or nothing property. Unquestionably the original researcher has hugely privileged access but that does not mean no one else can make any sense at all of fieldnotes etc (another intentional double negative). And in terms of *trust* a refusal to share raises the question of what they might be hiding while an openness to sharing implies of itself an degree of trustworthiness, even if the sharing may be far in the future. In other words, many of the reservations researchers may have about data sharing can be addressed by making very long embargoes the norm.

In publications from the harder sciences, issues of trust revolve around whether the published results and the text discussing them are trustworthy (T_3_). To help assess this access to the underlying data is helpful. However, other issues of trust accrue in qualitative social sciences before similar questions about the trustworthiness of publications can be asked. Usually tacit or unspoken, there are different questions of trust underlying consent giving and subsequent interactions: the respondents or interlocutors have to assess the trustworthiness of the researchers (T_1_): can these individuals and can the promises they make be trusted? These are different points to those about the trustworthiness of publications. This is fundamental since, of course, if participants really don’t trust the researchers then they will not continue and no material will be collected so no publications will result. If they have doubts they may still participate but may provide more or less unhelpful or misleading answers (Kuula, 2011:15). In some cases researchers acting in good faith have made promises they have not been able to keep. The clearest and most notorious instance of this is case of an oral history project about the “troubles” in Northern Ireland which was archived in Boston (United States). The researchers sought to resolve the issue of informant confidentiality by promising former participants in the conflict that their interview data would not be released until after the interviewee’s death. But the Northern Irish police used legal subpoenas to break these promises. The researchers had made promises in good faith that in the end legal process meant they were not able (were not allowed) to keep.^4^

In cases where consent is withheld, the potential participant has made a judgment that, for whatever reasons, the researcher cannot be trusted (T_1_), and no data is collected. Conversely, once someone has agreed to participate, the researcher has to make judgments about them: is their account to be trusted (T_2_)? This may change over time. A claim to have special access and insight may be initially accepted but then later changed as it becomes clear that the person in question does not have access and indeed may be widely regarded by others in the community as a notorious liar. Anthropologists have to manage being simultaneously credulous fools and ultimate cynics. I call this professional bad faith or adopting a position of ironic detachment; Johannes Fabian talks about “the duplicity without which ethnographic research would be impossible—a duplicity which makes us cross borders but not without establishing a record that lets us return to our professional roles and habits” (2008: 6).^5^ That said there is a lot that can be learned from liars such as what is deemed to be a plausible alternative version of events and motivations. Disputes in politics (and elsewhere) are revealing about process no matter what actually did occur or who did what to whom. Such material even if considered to be unreliable (T_2_) may yet be revealing hence useful and so is worth keeping.

When it comes to data sharing a researcher might well want to anonymize versions (I stress *versions*: not everything) of research material so it can be made accessible relatively soon after the data has been collected–paralleling the ways that the UK Census Office removes personal ID information from some material from recent censuses. Importantly, even though the publicly accessible version may be anonymized, if the key to the anonymization exists, then legally it remains personal data and subject to GDPR rules (and strictly should be called a pseudonymized version).^6^ I very much hope researchers retain the keys because the *longue-durée* view is, in my opinion, that the full data should be retained and made available but only in the long term. Perhaps, as with census data, this should default to being 100 years after collection. Even this, in some circumstances, may have to be restricted but for most material it is plausible to suggest that access could be enabled with only a very light administrative touch.^7^ The data may still be described as FAIR (meeting principles of findability, accessibility, interoperability, and reusability) only a delay has been introduced into the aspects of accessibility and reusability.

An example of why it is important to retain complete data records including the names may be found in the title of a book by Michel Foucault based on research in judicial archives.

I, Pierre Rivière, having killed my mother, sister and brother (1835) Published 1973 (filmed in 1976).

I think similar books and films should be possible in 100 years’ time with the full names given rather than:

I, Homme03061835, having killed my mother, sister and brother.

In other words I am arguing that any anonymization should be reversible. As already mentioned technically this is pseudonymization. Either a complete version of the material must be kept without pseudonyms, or a key retained to the pseudonyms used so that at a much later date the pseudonymised file can be re-edited to reinsert the actual names and other identifying information. (I suspect that in practical terms it is easier to keep two versions of the file but data managers may take a different view).^8^

## Ethical Contradictions

There are contradictions and conundrums in the ethical position of archiving. In related work Kirsten Bell (Bell, 2018 and Bell and Wynn, 2020) considers conflicting relationships and stances towards consent and participation. She discusses how researchers and those they work with can develop a “procedural ethics framework.” Neale and Bishop (2012) describe an example of such a framework for research archives. This addresses the conundrum that prior “informed” consent cannot be given for future research by unknown others asking unknown research questions.^9^ Bishop has also discussed other ethical concerns about qualitative data archiving and shown how these can be addressed, hence enabling data archiving and sharing (2009). In the short and medium terms archivists must act as trusted proxies,^10^ implementing the processes that the participants consented to, and serve as trusted gate-keepers to the data long into the future (with all the administrative and resource implications that go with this). Russell and Barley (2019) raise questions about “who owns research data” which has a more direct bearing on data archiving.

Consider the possibly conflicting, certainly different, responsibilities that researchers have towards:

- Informants
- Colleagues
- Informants at a later date and their descendants
- Future colleagues (including older versions of themselves)
- Research funders/institutions

These responsibilities and obligations point in different, mutually conflicting, directions. Respect for the privacy of individuals suggests anonymization, closure or not archiving (and data destruction), whereas respect for the descendants of those individuals in the distant future suggests openness and archiving for the long term.

**FIGURE 1:**
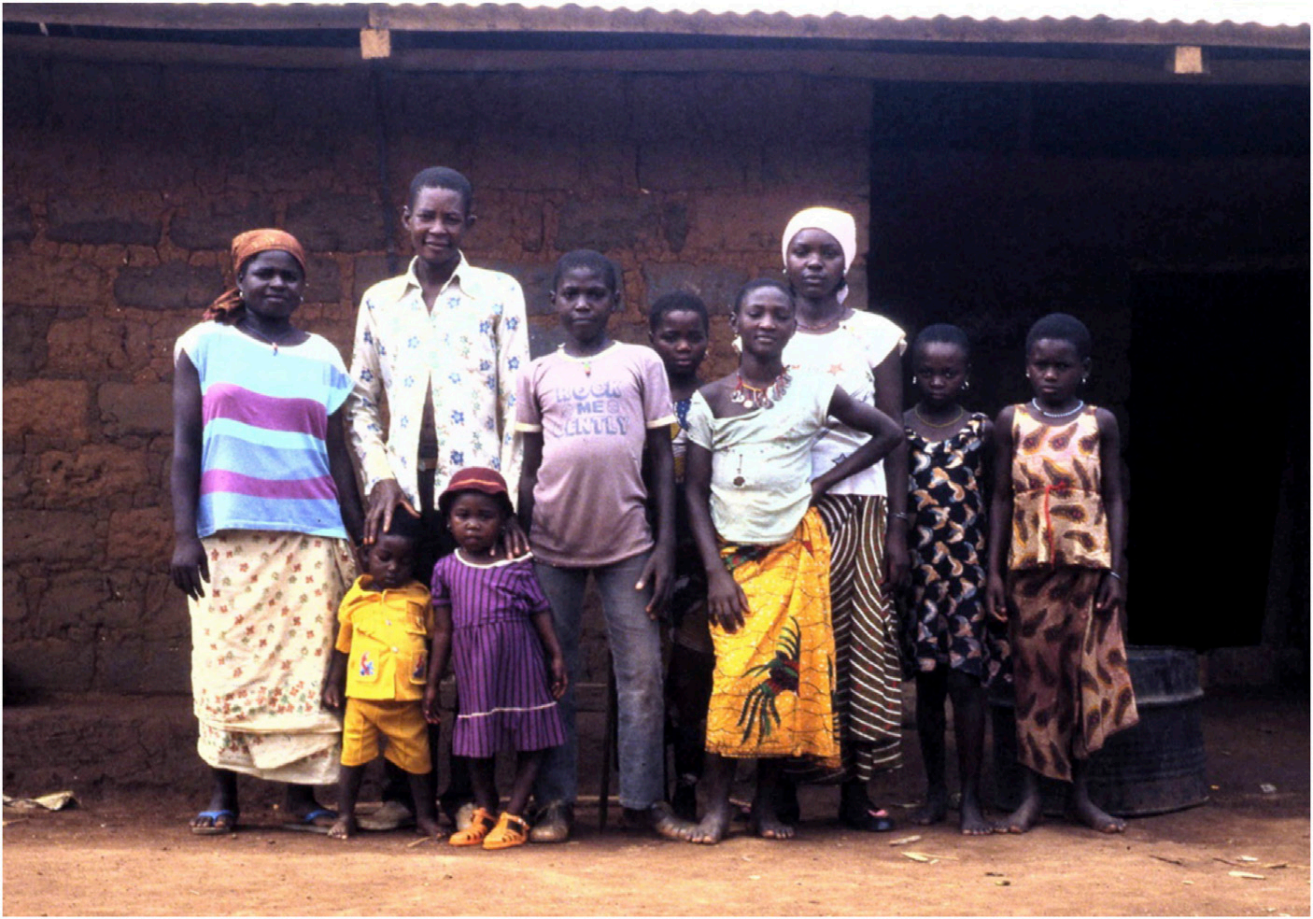
Caption: L-R: Barmi (alive 2018), Nde Donat (d 1987?), Ndignoua Salomon, Suzana Thia (alive 2018), Ngon Luise (died), Blandine (died), Dissi (Mougna’s child, died) Jacqueline (alive 2018), the two children in the front: the boy is Kounaka Fidèle (Salomon’s junior brother (alive 2018), and the girl is Mbitti Josephine (alive 2018). The names were provided in 2018 by Serge Donat, son of Blandine (photo David Zeitlyn Reference: 24_34. jpg 01/05/1986).

To make concrete the importance of preserving data such as photographs without blurring or other forms of anonymization let me give a clear example of why it would be unethical to destroy or fully anonymise data. Consider the following field photograph that I took in May 1986 (Figure 1).

Yanele Blandine, the girl in the white headscarf, is long dead. Her son, Serge Donat, had no photograph of his mother until recently, when I was able to send him a copy. Imagine his response if I had said either:

I took a photograph of your mother long ago but I cannot send you a copy because I destroyed it on completing my doctorate.
Or
I took a photograph of your mother long ago but I cannot send you a copy because I do not have her permission to share it.
Or
I took a photograph of your mother long ago but I can only send you a copy with her face blurred because I do not have her permission to share it.

I would suggest that all of these possible responses as suggested by the ethos of many ethics panels would be inhumanly rude and indeed would be deeply unethical.

There is a further almost banal point to be made—unnecessary for most of this readership but important nonetheless. Openness and Archiving does *not* mean:

1) giving free access to all comers
2) putting all material online without regulation (even for digital archives)

The UK Data Archive (RRID:SCR_014708 https://www.data-archive.ac.uk) is a digital repository for social science data. It includes quite a lot of anthropological material, and other qualitative data. It has developed ways of working with the sorts of unstructured material that anthropologists tend to produce. Access is not open in the sense of being uncontrolled: users have to register and in some cases *they* have to give a form of consent,^11^ so they enter into the same sort of relationships of trust with the informants that the original researcher did. Moreover, material placed into the archive can in some circumstances be embargoed (usually only for a relatively few years). However, the UK Data Archive has also worked with the UK Census to develop protocols (and technical solutions) for “Secure Access Points” which enable remote access to highly sensitive datasets in constrained and controlled fashion. To summarize: Digital Archives can have a wide range of different types of access—ways that have more resemblance to access to physical archives than most digital evangelists might imagine. My suggestion is that a default for qualitative data might be 100 years, then in some unusual cases longer embargoes could still be called for, in many others much shorter ones. Even within the embargo period tightly controlled access could be arranged (again as is currently possible for census data). This is to suggest that what could be called “archival hesitancy,” a suspicion about data archiving (sharing) among qualitative social scientists, can be, partly, addressed by shifting to a census-style default from which one could discuss variations. Such an environment would only be possible if research funders allow data to be archived under census-type protocols *and* if archiving services such as the UK Data Archive are given the resources to enable this.

In short anthropology and its fellow subjects can manage to resolve the injunctions of morality and ethical responsibility to our research collaborators with the conflicting pressures to be open and share our data by resolving to be open in ways that parallel how the census is open. A recent example of how openness can be achieved over a century or more is the publication of Alfred Haddon’s diaries (Herle and Philp 2021). This was undertaken in collaboration with the descendants of the people Haddon worked with in the Torres Straits in 1888 and 1898. This is the timescale we need to start thinking about, this is the timescale that openness in anthropology and cognate disciplines can accept as ethically consistent with the promises we make. The challenge now is to resource infrastructures that can accommodate ethical complexity: to enable us to be open but not quite yet (Parry and Mauthner, 2004; Bishop, 2009; Blisset, unpublished^12^).

## Footnotes

1 This has led one pseudonymous author to suggest they should be coauthors. See Luther Blissett mss.

2 It may even apply to those in humanities discussing the work of living authors: I suspect few in disciplines such as English etc have considered the relevance of GDPR and data sharing for their research.

3 Pursuing this is in danger of taking me off topic so I will not further discuss wider purposes and limits of data sharing here.

4 See Lowman and Palys (2013) as well as news coverage such as https://www.bbc.co.uk/news/uk-northern-ireland-27238797 and also https://www.irishtimes.com/news/crime-and-law/attempt-to-access-former-ira-man-s-boston-college-tapes-replete-with-errors-court-told-1.3357750 Both accessed 26 Sept 2018.

5 The ambiguities involved have also been discussed as the part of the dilemmas of ‘insider/outside’ positionality (Zavella 1996).

6 Note that if truly anonymized and there is no key to reverse the process then they are no longer personal data and therefore not subject to GDPR regulations.

7 Users may still have to register and the archives may wish their role to be acknowledged in any resulting publications.

8 A Frontiers editor has suggested that cryptographic protocols or infrastructures such as the Blockchain could provide mechanisms for deterministically reversible psuedonymisation (i.e. reversible at a pre-destined point in time based on either computational difficulty to crack anonymity or on a smart contract). I find this an intriguing suggestion but fear it may involve too complex a bet on future infrastructures to achieve wide take up. Keeping two copies, only one of which is pseudonymised seems simpler.

9 An example might be a linguist studying the phonetics of vowel quality in interview sound recordings. The linguist might be interested because the interviewer had recorded where participant and their grand-parents lived but be entirely uninterested in the content of the discussion that was recorded.

10 Hence the importance of certification schemes for repositories such as Core Trust Seal (RRID:SCR_021679 https://www.coretrustseal.org/) by which they can be assessed as ‘Trusted Digital Repositories’.

11 Those reusing the data have to sign a User Agreement from the archive with binding terms and conditions that define what they can, and cannot, do with the data they access. This has parallels with the original consent process.

12 Blissett, L. 2006. The Hau of the Paper and Dividual Authors: Reimagining Authorship in Anthropology. Unpublished manuscript.
